# Strychnia Taken by Mistake for Morphia

**Published:** 1848-01

**Authors:** 


*Strychnia taken by mistake for Morphia.*—An accident similar to that of Dr. Warner, of which an account was published in our Nov. number, lately occurred at Springfield, Ohio. Dr. John Patton, having procured a bottle of Morphia, and one of strychnia, both supposed to contain Morphia, took a small quantity of the Strychnia on the point of a pen-knife, and died soon afterward. These accidents enforce the importance of great carefulness on the part of Apothecaries and Practitioners.

